# Rate of Complications and Subsequent Shoulder Complaints for Non-operatively Managed Pediatric Proximal Humerus Fractures

**DOI:** 10.3389/fsurg.2020.00048

**Published:** 2020-07-31

**Authors:** Courtney Baker, Noelle Larson, William Shaughnessy, Anthony Stans, Todd Milbrandt

**Affiliations:** Mayo Clinic, Rochester, MN, United States

**Keywords:** pediatric proximal humerus fractures, minimally displaced, non-operative management, resource utilization, long term follow up

## Abstract

**Purpose:** The purpose of the study was to describe the early complications and delayed shoulder complaints of non-displaced or minimally displaced pediatric proximal humerus fractures treated non-operatively.

**Methods:** Retrospective review of all pediatric proximal humerus fractures at a single institution from 2001 to 2016. Inclusion criteria were: AP and axillary radiographs upon presentation and final follow up, one follow up appointment, either a non-displaced or minimally displaced fracture, and open physis. Exclusion criteria were: pathologic fractures, re-fractures, bone metabolic disorders. Patient demographics, injury characteristics, radiographic measurements and clinical exam findings were reviewed. Delayed shoulder complaints were defined as a visit to any provider for an ipsilateral shoulder or arm complaint after final scheduled fracture appointment.

**Results:** Sixty-nine of 177 total pediatric proximal humerus fractures met inclusion criteria. Mean age was 10 years (SD = 3.4). Sixty-five had angulation <20 degrees. Median time to last scheduled follow up was 1.4 months (Interquartile range 0.8–1.4). At last scheduled follow up, 9 (13.0%) fractures had an altered exam. One (1.4%) fracture had a complication of a fall and re-fracture. Extraphyseal fractures were more likely to increase in angulation at short term follow up, but had no association with short or long term complications. No patient initially treated with non-operative management subsequently underwent operative treatment. There were three presentations (4.3%) to health professionals for subsequent shoulder complaints; one was treated with short courses of physical therapy and the other two were simply observed.

**Conclusions:** Non-displaced or minimally displaced proximal humerus fractures treated non-operatively sustain rare short or long term complications with no cross over to operative management. Once non-operative management is pursued, these fractures may be amenable to surveillance with primary care or allied orthopedic staff after initial evaluation by pediatric orthopedic providers.

**Level of Evidence:** Level III retrospective cohort study.

## Introduction

Pediatric proximal humerus fractures account for roughly 2% of all pediatric fractures (1, 2). The proximal humerus is responsible for ~80% of longitudinal growth of the humerus (2–5), and the shoulder is a highly mobile structure comprised of three joints and one articulation. Multiple authors have proposed varying indications for surgical management based on acceptable limits for angulation and displacement depending on the patient's age (2, 5–8). However, the majority of proximal humerus fractures are not severely displaced or angulated and, given the remarkable remodeling potential and mobility of the shoulder joint, are consequently treated non-operatively (2–5). There remains a paucity of reports characterizing long term follow up for patients treated non-operatively, and it is not known whether those fractures treated non-operatively become problematic years after the fracture has healed leading to delayed shoulder pathology.

The purpose of this study was to evaluate the outcomes of non-displaced or minimally displaced pediatric proximal humerus fractures treated non-operatively both in terms of early complications and delayed shoulder complaints. Our hypothesis was that non-displaced or minimally displaced proximal humerus fractures would suffer few early complications and have low rate of delayed shoulder complaints. The primary outcome was presentation to any healthcare provider with an ipsilateral shoulder complaint after completion of scheduled fracture care. Secondary outcomes included abnormal exam findings at final scheduled follow up, re-fracture, or operative intervention for any reason in this group of patients treated non-operatively.

## Materials and Methods

This was an IRB approved, retrospective review of all pediatric proximal humerus fractures at a single institution from 2001 to 2016. Inclusion criteria were: AP and axillary radiographs upon presentation and final follow up, one follow up appointment, either a non-displaced or minimally displaced fracture [<5 mm (Neer and Horwitz classification I)], and open physis on radiographs. Exclusion criteria included pathologic fractures, re-fractures or metabolic/pathologic disorders contributing to fracture etiology. Patient demographics (sex, age, and laterality), injury characteristics (mechanism, open, vascular or nerve injury), radiographic measurements (as below), clinical exam findings at presentation and each follow up visit, and treatment management were recorded.

All non-displaced or minimally displaced fractures were treated in a sling or shoulder immobilizer at the time of presentation and made non-weight bearing until clinic follow up with pediatric orthopedics. Activity was advanced during clinical follow up based on clinical exam, radiograph assessment, and time from injury.

Fracture radiographs were evaluated for involvement of the physis, Salter Harris classification (as appropriate), displacement on both AP and axillary view, and angulation on AP view based on method described by Burke et al. (9). All radiographs were measured by a single author (CEB). Clinical evaluations were reviewed and presence of pain, diminished range of motion, or weakness on exam within 6 weeks of injury were considered “abnormal exams” rather than complications as such symptoms are expected during fracture healing.

The primary outcome was presentation to any healthcare provider with an ipsilateral shoulder complaint after completion of scheduled fracture care. Secondary outcomes included abnormal exam findings at final scheduled follow up, re-fracture, or operative intervention for any reason in this group of patients initially treated non-operatively.

Patient records were reviewed from injury through 2018 for presentation to any provider within the institution's multi-state system of clinics (family medicine, pediatric or adult orthopedics, sports medicine, physical therapist, etc.) for an ipsilateral shoulder or arm complaint after final fracture monitoring appointment. The reason for presentation and management was recorded. These individuals' final clinical exam was then reviewed for changes in radiographic parameters or exam abnormalities. The study was conducted at the only pediatric referral center for the area and thus patients who did not present for further follow up were assumed to have a clinically acceptable result without significant shoulder complaint (10).

Statistical analysis was carried out in JMP (JMP Pro 13.0; Cary, NC). Basic statistical tests and Wilcoxon Rank Sum test for significance were conducted as indicated.

## Results

**Figure 1:**
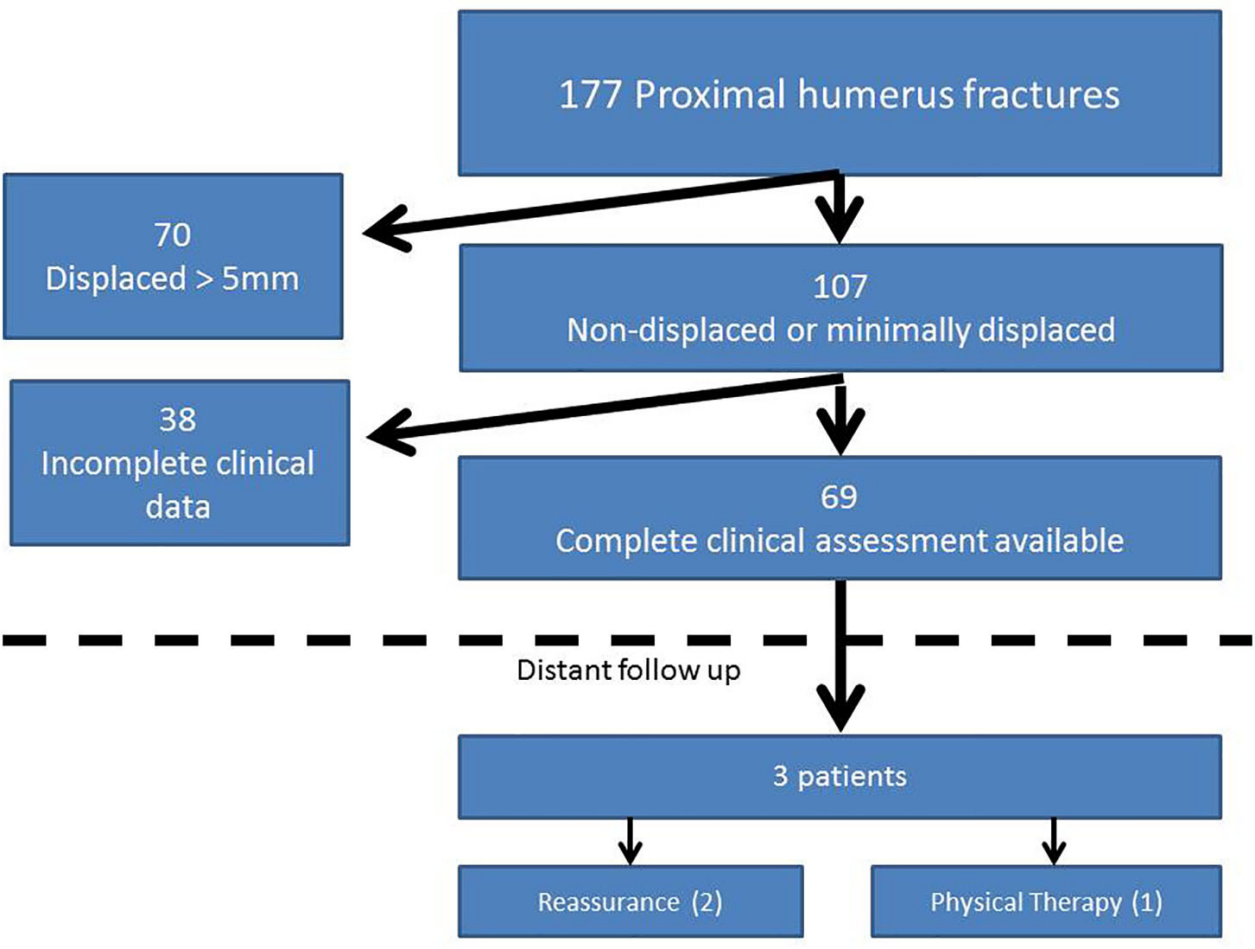
Subject inclusion breakdown.

Basic patient and injury demographics can be found in Table 1. There were 177 total pediatric proximal humerus fractures during the 15 year period, 151 treated non-operative and 26 treated operatively. One hundred seven fractures were either non-displaced or minimally displaced. Sixty-nine fractures had complete radiographic assessment and minimum one clinical follow up appointment and were included in the study (Figure 1). Mean age was 10 years (standard deviation = 3.4). Thirty-seven fractures did not involve the physis (53.6%), six were Salter Harris type 1 (8.7%), and 26 were Salter Harris type 2 (37.6%).

**Table 1:** Demographics and characteristics of non-displaced or minimally displaced pediatric proximal humerus fractures.

	***N* (% of total)**	**Mean**	**Standard deviation**
Total cases	69 (100)		
Age at injury, years		10	3.4
**Sex**			
M	33 (47.7)		
F	36 (52.2)		
Open injury	0		
**Injury mechanism**			
Fall	57 (82.6)		
Motor Vehicle Accident	4 (5.8)		
Other	8 (11.6)		
Concomitant injury	7		
Nerve injury	0		
**Salter Harris classification**			
Uninvolved physis	37 (53.6)		
1	6 (8.7)		
2	26 (37.6)		
3	0		
4	0		
**Injury Angulation**			
<20	65 (94.2)		
20–40	4 (5.8)		
40+	0		
**Neer and Horwitz classification**			
Non-displaced	17 (24.6)		
1	52 (73.4)		

Of the 69 non-displaced or minimally displaced fractures, seven patients had concomitant injuries including: distal radius fracture (*N* = 3), acromion fracture (*N* = 2), loss of consciousness (*N* = 1), and forehead bruising (*N* = 1). On AP radiographs, 65 had angulation <20 degrees and four had angulation between 20 and 40 degrees. Median time to last scheduled follow up was 1.4 months (Interquartile range 0.8–1.4) (Table 2). At last scheduled follow up, 9 (13.0%) fractures had an altered exam: five had reduced range of motion, three had pain, one had reduced strength (Table 3). In patients with reduced range of motion, minimum 90 degrees of forward flexion was achieved at 3 weeks from injury, except for one patient who remained strictly immobilized until follow up and had only 30 degrees forward flexion. The patient was taught shoulder range of motion exercises and did not return for subsequent follow up. Three patients (4.3%) had pain documented on exam associated with near full motion at 3, 3, and 6 weeks from injury, respectively. One patient (1.4%) had documented 3/5 strength in deltoid, biceps, triceps with guarding on exam at 6 weeks from injury. One (1.4%) fracture had a complication of a fall and re-fracture.

**Table 2:** Short term and distant follow up of non-displaced or minimally displaced pediatric proximal humerus fractures.

	***N* (% of total)**	**Median**	**Interquartile range**
**Time to follow up**			
Last scheduled visit, months	69 (100)	1.4	0.8–1.4
Distant follow up, months	3 (4.3)	40.7	11.6–55.1

**Table 3:** Outcomes of non-displaced or minimally displaced pediatric proximal humerus fractures.

	***N* (% of total)**	**Mean**	**Standard deviation**
**Treatment**			
Non-op	69 (100)		
**Follow up angulation (degrees)**			
<20	48 (67.6)		
20–40	19 (26.8)		
40-60	2 (2.8)		
**Change in angulation (degrees)**			
Physeal fractures	32 (46)	1.6	9.0
Extraphyseal fractures	37 (54)	9.6*	12.7
**Complication at last visit**			
Fall and re-fracture	1 (1.4)		
Delayed operative intervention	0 (0)		
**Abnormal final exam**	9 (13.0)		
Range of motion	5 (7.2)		
Pain	3 (4.3)		
Strength	1 (1.4)		

* *P < 0.01*.

**Figure 2:**
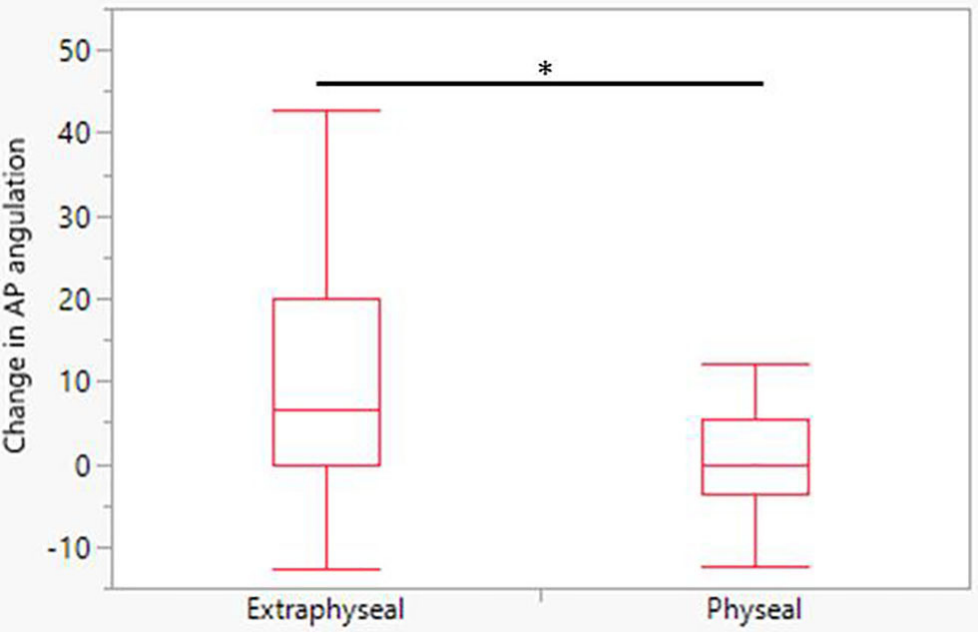
Change in AP radiograph angulation at final follow up. Extraphyseal fractures have significantly greater change in angulation measured at final follow up. * = *P* < 0.01.

Final radiographic assessment demonstrated 48 fractures had angulation <20 degrees, 19 had angulation between 20 and 40 degrees and two had angulation 40–60 degrees. Change in AP angulation at final follow up was significantly greater in extraphyseal fractures compared to physeal fractures,1.6 and 9.6 degrees for physeal and extraphyseal fractures, respectively, and significantly different (*P* < 0.01) (Table 3, Figure 2). There was no association with change in AP angulation and age (data not shown). No fractures had greater displacement than presenting injury films. There were no cases of premature physeal closure.

No patient initially treated with non-operative management was subsequently taken for operative intervention based on clinical or radiographic examination.

In the 69 non-displaced or minimally displaced fractures included in the study, there were three presentations to health professionals for subsequent shoulder complaints (4.3%, Figure 1) at 11.6, 40.7 and 55.1 months from original injury, respectively, none of whom had altered exam at last scheduled follow up. All three patients presented with chief complaints of shoulder pain with activity. The patients' proximal humerus angulation at initial presentation, last scheduled follow up, and subsequent presentation, respectively, were: 28, 26, 13; 3, 20, 3; 13, 11, 12 (degrees). One of these patients was treated with short courses of physical therapy and the other two were simply observed. None required operative intervention.

## Discussion

Pediatric proximal humerus fractures are rare occurrences and lend themselves to non-operative management because of the robust growth potential of the proximal humerus and mobility of the shoulder. The purpose of this study was to evaluate the outcomes of non-displaced or minimally displaced pediatric proximal humerus fractures treated non-operatively both in terms of early complications and delayed shoulder complaints. The hypotheses were confirmed: 1.4% early complication rate, 13% abnormal exam at final scheduled follow up, 4.3% delayed shoulder complaints—none of whom required more than a course of physical therapy. This data provides evidence-based rates for counseling patients and their families who sustain non-displaced or minimally displaced proximal humerus fractures on long-term outcomes of non-operative management.

In deciding management for these injuries, much discussion has focused on severe fractures in adolescents with less opportunity for remodeling. It is widely accepted that children and adolescents with Neer and Horwitz grade I and II proximal humerus fractures should be treated non-operatively (11, 12). These are typically managed in a sling of some fashion for 3 to 4 weeks. Non-operative management is not recommended even for minimally displaced fractures in the context of open injuries, vascular injury or polytrauma (7). In our cohort there were no open injuries, and eight with concomitant injuries. Varying criteria for surgical intervention based on angulation exist. In general, these operative indications are progressive reductions in acceptable angulation based on age (2, 5–8). A lower-threshold indication for surgery is limiting acceptable angulation to 30 degrees in patients older than 10 years (5). A higher-threshold surgical indication is accepting up to 45 degrees angulation in those older than 12 years. In either case, our treatment decisions at the time of injury are consistent with current accepted practice, and the study makes no assessment on criteria for selecting operative vs. non-operative management. Rather it informs providers based on the outcomes once pursuing non-operative management.

Regarding early complications, outcomes for proximal humerus fractures are generally favorable. In a 2010 systematic review, at final follow up of non-operatively treated patients, 6% had restricted range of motion, 7% had pain, and 4% had shortening (8). Bahrs et al. found no complications and Constant Score of 100 in 10 non-displaced fractures at average follow up of 39 months (5). Our cohort sustained a single complication of fall and re-fracture and no individual who was managed non-operatively subsequently required an operative intervention. Our results for abnormal final exam showed altered range of motion (7.2%), pain (4.3%), and or diminished strength (1.4%) which are consistent with previously published results. The data herein confirm that if final scheduled follow up is within 6 weeks from injury, a minority of patients will still have an abnormal exam. The question this study aims to elucidate is how many of these patients who we presume will return to normal function will actually present at a distant time with ipsilateral shoulder complaints. Our cohort's incidence of delayed shoulder complaints was 4.3%. None of these patients who re-presented had an abnormal exam at final scheduled follow up.

At final scheduled follow up 17 fractures' angulation had increased from <20 to >20 degrees, and extraphyseal fractures demonstrated a significant increase in angulation compared to physeal fractures (Table 3, Figure 2). This may be a consequence of insufficient immobilization after injury or possibly more accurate radiographic assessment once the initial trauma has passed. Though it was common in this cohort for fracture angulation to increase after presentation, no patient re-presented with a chief complaint related to malunion or angulation. It is notable that despite persistent and sometimes worsened angulation, the majority of patients had painless, full range of motion and intact strength. It is uncertain whether the increase in angulation at short term follow up for extraphyseal fractures has any clinical significance. Future studies with longer term radiographic assessment are required to evaluate whether short term increase in angulation resolves or is maintained but functionally insignificant.

Gladstein et al. (13) reviewed 239 patients who presented to a tertiary referral center with proximal humerus fractures over a 5 year period to evaluate whether follow-up radiographs changed management in these patients. Two hundred twenty-five were managed non-operatively with 1 requiring a subsequent epiphysiodesis at 1 year for partial growth arrest. They concluded that non-operative treatment seldom results in subsequent displacement that warrants operative treatment. Our data further support these findings that it is exceedingly rare for proximal humerus fractures determined to be appropriate for non-operative management have the need for further intervention in the absence of new trauma. Gladstein et al. also concluded that there is little utility to routine use of post-injury radiographs in follow-up of these patients. Our study similarly demonstrated no changes to the management of patients based on follow up radiographs and, while not directly tested given its retrospective design, this questions the value of routine radiographs in the absence of repeat injury or worsening exam findings. One wonders whether these patients can be spared routine monitoring radiographs, similar to a recommendation following pediatric supracondylar humerus fractures (14).

The optimal balance of education and clinical testing provided during pediatric fracture follow up appointments and, more importantly, by which providers remains to be determined. There is an ongoing discussion of which pediatric fractures need follow up with orthopedic surgeons and which are safely managed by primary care or emergency medicine providers. Morris et al. found there was a significant opportunity cost to pediatric fracture follow up appointments and only 25% of appointments involved changes in management; however, 99% of parents found the “visit was of value” (15). From a resource perspective, multiple previous studies have called into question the amount of follow up necessary for certain pediatric fractures including distal radius fractures and clavicle fractures (10, 15, 16). Given the low complication rate and incidence of subsequent shoulder complaints in ours and others' studies, it raises the question of whether non-displaced or minimally displaced pediatric proximal humerus fractures may be safely managed by primary care physicians or orthopedic mid-level providers after initial assessment by a pediatric orthopedic specialist. Based on the current body of literature, the authors propose that these fractures be considered alongside pediatric distal radius buckle fractures and other minor fractures that are already primarily managed by emergency department, primary care providers, or orthopedic-trained advanced practitioners (16, 17). Future prospective studies are required to ensure the safety and efficacy of such practice. Coordinated care pathways between the emergency department, pediatric orthopedic surgeons and primary care providers may provide the most efficient surveillance system for these fractures while minimizing costs to the patient and health system. As with other minor fractures, education regarding the natural history and outcomes of non-operative management of these fractures may make primary care providers more comfortable with managing these injuries (17, 18). The authors recognize that, despite appropriate counseling, some patient families and/or primary care providers will still request or require continued follow up with a pediatric orthopedic surgeon to provide reassurance even in these benign cases.

This study is not without bias or limitation. It was conducted at a single institution which may limit its generalizability to other centers and populations. The principle limitation is the retrospective nature and assumption that all subsequent shoulder or arm complaints were identified. However, the investigation was conducted in a relatively stable population over a long period of time within the only pediatric tertiary referral center and major adult orthopedic referral center for the surrounding area. We believe this scenario provides the best opportunity to capture as many subsequent shoulder complaints as possible given the retrospective investigation. A prospective study with directed, long term follow up would better ensure all subsequent shoulder complaints were captured. In addition 36% of non-displaced or minimally displaced fractures were excluding for incomplete radiographic assessment which may have introduced selection bias. Radiograph assessment of angulation and displacement in these fractures is frought with challenges based on patient compliance and anatomic limitations and introduces assessment bias to the results given the retrospective design. This makes characterization of proximal humerus fractures challenging but inherent to all investigations of this topic, particularly retrospective studies. Radiographic assessment was standardized according to the technique described by Burke et al. (9) and completed by one reviewer to maximize consistency. Additionally, given the retrospective nature of the study, standardization of care provided was not ensured. Prospective studies that obtain dedicated long term radiographic and clinical follow are required to confirm the low rate of complications and subsequent shoulder complaints.

In conclusion, in a large cohort of pediatric proximal humerus fractures, the majority were either non- or minimally displaced. Early complications were rare and altered exam at last scheduled visit was not a harbinger of distant follow up for shoulder complaints. Routine follow up radiograph assessment did not change management of any patients. 4.3% of patients presented for ipsilateral shoulder complaints well after final scheduled follow-up, and these were addressed successfully with a short course physical therapy. The study adds to the growing body of evidence which demonstrates those non-displaced or minimally displaced proximal humerus fractures amenable to non-operative management suffer rare complications in the absence of repeat trauma, and suggests these fractures may be safely treated with surveillance by primary care or allied orthopedic staff after initial consultation with a pediatric orthopedic provider.

## Data Availability Statement

The datasets generated for this study are available on request to the corresponding author.

## Ethics Statement

The studies involving human participants were reviewed and approved by Mayo Clinic IRB. Written informed consent from the participants' legal guardian/next of kin was not required to participate in this study in accordance with the national legislation and the institutional requirements.

## Author Contributions

CB and TM: study design. CB, NL, WS, AS, and TM: data acquisition, analysis and interpretation of data, and drafting and revision of manuscript. All authors contributed to the article and approved the submitted version.

## Conflict of Interest

The authors declare that the research was conducted in the absence of any commercial or financial relationships that could be construed as a potential conflict of interest.
